# Expansion and stress responses of the AP2/EREBP superfamily in cotton

**DOI:** 10.1186/s12864-017-3517-9

**Published:** 2017-01-31

**Authors:** Chunxiao Liu, Tianzhen Zhang

**Affiliations:** National Key Laboratory of Crop Genetics & Germplasm Enhancement, Cotton Research Institute, Nanjing Agricultural University, Nanjing, 210095 Jiangsu Province People’s Republic of China

**Keywords:** *Gossypium*, Polyploid, Duplicated genes, Homoeologous genes, Gene expansion, Stress response

## Abstract

**Background:**

The allotetraploid cotton originated from one hybridization event between an extant progenitor of *Gosssypium herbaceum* (A_1_) or *G. arboreum* (A_2_) and another progenitor, *G. raimondii* Ulbrich (D_5_) 1–1.5 million years ago (Mya). The APETALA2/ethylene-responsive element binding protein (AP2/EREBP) transcription factors constitute one of the largest and most conserved gene families in plants. They are characterized by their AP2 domain, which comprises 60–70 amino acids, and are classified into four main subfamilies: the APETALA2 (AP2), Related to ABI3/VP1 (RAV), Dehydration-Responsive Element Binding protein (DREB) and Ethylene-Responsive Factor (ERF) subfamilies. The *AP2/EREBP* genes play crucial roles in plant growth, development and biotic and abiotic stress responses. Hence, understanding the molecular characteristics of cotton stress tolerance and gene family expansion would undoubtedly facilitate cotton resistance breeding and evolution research.

**Results:**

A total of 269 *AP2/EREBP* genes were identified in the *G. raimondii* (D5) cotton genome. The protein domain architecture and intron/exon structure are simple and relatively conserved within each subfamily. They are distributed throughout all chromosomes but are clustered on various chromosomes due to genomic tandem duplication. We identified 73 tandem duplicated genes and 221 segmental duplicated gene pairs which contributed to the expansion of AP2/EREBP superfamily. Of them, tandem duplication was the most important force of the expansion of the B3 group. Transcriptome analysis showed that 504 *AP2/EREBP* genes were expressed in at least one tested *G. hirsutum* TM-1 tissues. In *G. hirsutum*, 151 non-repeated genes of the DREB and ERF subfamily genes were responsive to different stresses: 132 genes were induced by cold, 63 genes by drought and 94 genes by heat. qRT-PCR confirmed that 13 *GhDREB* and 15 *GhERF* genes were induced by cold and/or drought. No transcripts detected for 53 of the 111 tandem duplicated genes in TM-1. In addition, some homoeologous genes showed biased expression toward either A-or D-subgenome.

**Conclusions:**

The *AP2/EREBP* genes were obviously expanded in *Gossypium*. The *GhDREB* and *GhERF* genes play crucial roles in cotton stress responses. Our genome-wide analysis of *AP2/EREBP* genes in cotton provides valuable information for characterizing the molecular functions of *AP2/EREBP* genes and reveals insights into their evolution in polyploid plants.

**Electronic supplementary material:**

The online version of this article (doi:10.1186/s12864-017-3517-9) contains supplementary material, which is available to authorized users.

## Background

The APETALA2/ethylene-responsive element binding protein (AP2/EREBP) superfamily is one of the largest and specific transcription factor (TF) families in plants. Members are defined by their AP2/ERF domain, which comprises about 60 to 70 amino acids [1–3]. They play essential roles in plant growth, development and responses to various environmental stresses, including cold, heat, drought, high salinity and pathogen infection, by directly responding to stresses or regulating the expression of downstream target genes [4–7]. The AP2/EREBPs have also been implicated in different hormones-related signal transduction pathways including ethylene, abscisic acid (ABA), cytokinin and jasmonate (JA) [2, 8–10].

Since the release of the whole-genome sequences of many plant species, the AP2/ERFEBP superfamily has been successfully identified and investigated in a variety of plant species, such as Arabidopsis (147) [11], poplar (200) [12], soybean (98) [13], tomato (85) [14], rice (163) [15], potato (155) [16], *Medicago truncatula* (123) [17] and *Brachypodium Distachyon* (149) [18]. All these *AP2/EREBP* genes are characterized by one or two AP2 DNA binding domains, which consist of a three-stranded anti-parallel β-sheet and an α-helix [19]. In the model plant Arabidopsis, the 147 *AP2/EREBP* genes are divided into four subfamilies: the APETALA2 (AP2), Dehydration-Responsive Element Binding protein (DREB), Ethylene-Responsive Factor (ERF) and Related to ABI3/VP1 (RAV) subfamilies [20]. The AP2 subfamily members, which contain two AP2 domains, have important functions in the regulation of plant growth and development [21–26]. The RAV subfamily transcription factors, which possess a single AP2 domain and an additional B3 domain [27], as well as a DNA-binding domain commonly found in other TFs [28], play significant roles in the regulating expression of target genes in response to ethylene, brassinosteroids and environmental stresses [29–32]. The ERF and DREB subfamily members, which contain a single conserved AP2 domain and comprise the largest groups in the AP2/EREBP superfamily, play critical roles in stress responses and in a variety of other plant processes. Additionally, a fifth group comprises all other AP2/EREBPs not assigned to the other four groups: there is only one gene (At4g13040) in this group in Arabidopsis [11].

Sequences contained only one AP2 domain had the greatest number of members in the AP2/EREBP superfamily, which are further divided into two major subfamilies, the ERF and DREB subfamilies [33]. The ERF subfamily members directly bind to GCC-boxes (AGCCGCC) and regulate the expression of pathogenesis-related (PR) genes. In addition, the ERFs are involved in hormone signaling pathways, such as the ethylene, JA, and salicylic acid (SA) pathways, which are important for plant development and stress responses [34–37]. The DREB subfamily members bind to the dehydration-responsive element/C-repeat, A/GCCGAC (DRE/CRT) elements, which are present in stress-responsive genes such as *RD29* and *COR15* genes [38, 39]. They regulate stress-responsive genes in response to various abiotic stresses, including cold, heat, drought and salinity [28, 40, 41]. In Arabidopsis the ERFs and DREBs were further divided into 12 groups, A1-A6 (the DERB subfamily) and B1-B6 (the ERF subfamily) by Sakuma et al. [33]. Recently, a new group B7 was identified in the ERF subfamily in rice and *Brachypodium Distachyon* [15, 18], indicating that the ERF genes may have further functions in plants.

Cotton is one of the most important economic crops worldwide and provides the world’s leading natural textile fiber and considerable amounts of edible oil. Studies have shown that allotetraploid *Gossypium* species were formed by a polyploidization event that occurred 1 ~ 1.5 million years ago (Mya), involving an A-genome species *G. arboreum* and a paternal D-genome species *G. raimondii* [42]. As a young allotetraploid plant, *G. hirsutum* is an excellent model plant for research into the functional divergence and evolution of duplicated genes. Since the release of the whole-genome sequences of the diploid cotton *G. raimondii* [43] and the allotetraploid cotton *G. hirsutum* (TM-1) [42], genome-wide analysis of *AP2/EREBP* genes is possible, and will help to elucidate their regulatory functions in plant growth, development and, in particular, stress responses. Although many other gene families and some members of the *AP2/EREBP* genes have been studied in cottons, the AP2/EREBP superfamily remains largely unexplored in cotton. Genomic analysis is an effective means of transferring knowledge from one taxon to another [44], and can facilitate our understanding of the expansion and functions of the *AP2/EREBP* superfamily genes in the evolution of cotton. In this study, we performed a comprehensive analysis of the AP2/EREBP superfamily in cotton, including phylogenetic tree, chromosomal localization, gene structure, gene expansion, and synteny analyses, as well as investigations into the expression profiles of these genes in various tissues and their expression patterns under different stresses. The results will help future investigations aimed at the functional characterization of stress tolerant *AP2/EREBP* genes, and can be utilized in the genetic improvement of cottons and studies into their genetic evolution.

## Results

### Genome-wide identification of the AP2/EREBP superfamily in cotton

The allotetraploid cotton species appeared about 1–1.5 Mya through the hybridization of a maternal A-genome species and a paternal D-genome species [42]. The whole genome sequence scaffolds of two sequenced cotton species (*G. raimondii* [43] and *G. hirsutum* acc. TM-1 [42]) were used for the genome-wide exploration of the AP2/EREBP gene family in *Gossypium*. Using the Hidden Markov Model (HMM) (HMMER v3.0) method with data from a query on the AP2/EREBPs family (PF00847), we searched the protein databases, and obtained a total of 269 AP2/EREBPs in *G. raimondii* after confirming the presence of the ‘AP2’ domain (Additional file 1: Table S1). As a young polyploidy species, genes from parental genomes were mostly retained in the *G. hirsutum* subgenomes [42]. In this study, we identified 504 AP2/EREBPs, 252 in the A subgenome and 252 in the D subgenome, in *G. hirsutum* acc. TM-1 (Additional file 2: Table S2). The homologous between *G. raimondii* and *G. hirsutum* showed high similarity and clustered together on the phylogenetic tree with one gene from *G. raimondii* and two from *G. hirsutum* (Additional file 3: Figure S1). The number of AP2/EREBPs in allotetraploid cotton has nearly doubled compared to that in diploid cotton, although a handful of homologs have been lost. We named the *AP2/EREBP* genes in *G. hirsutum* corresponding to their relationships to that of in *G. raimondii*, and marked A or D after each gene name to represent these genes belonging to A-subgenome or D-subgenome.

### Classification and phylogeny of the *AP2/EREBP* genes in *Gossypium*

Based on sequence similarities, the composition of domains and the number AP2 domain, the 269 *AP2/EREBP* genes in *G. raimondii* were divided into four groups. Specifically, four genes were grouped as outsiders with relatively complex sequences; 32 genes containing two complete AP2 domains were assigned to the AP2 subfamily; 11 genes had a single AP2 domain and a single B3 domain and were classified into the RAV subfamily; and the remaining 222 genes carried a single AP2/ERF domain and were assigned to the DREB/ERF subfamilies. The DREB/ERF subfamily members were further divided into DREB (80) and ERF (142) subfamilies based on sequence similarities (Additional file 4: Figure S2). Excluding the four outsider genes, the 265 *AP2/EREBP* genes were named *GrAP2-1-32*, *GrDREB1-80*, *GrERF1-142* and *GrRAV1-11*, respective to their subfamilies (Additional file 1: Table S1).

**Fig. 1:**
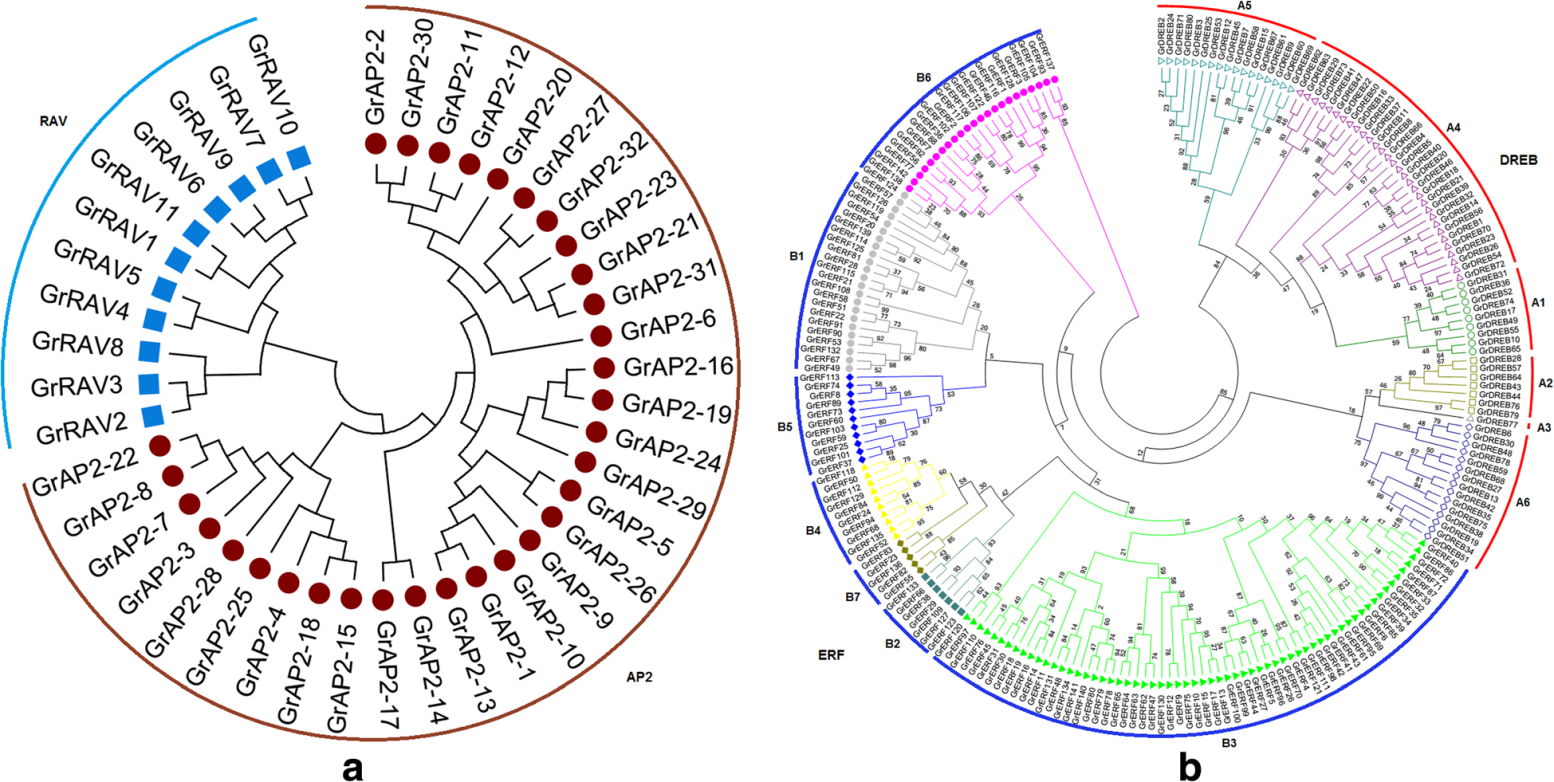
Phylogeny trees of the *G. raimondii* AP2/EREBP superfamily genes. **a ﻿**Phylogeny of the *G. raimondii *AP2 (APETALA2) and RAV (Related to ABI3/VP1) subfamily proteins based on their conserved domain sequences. **b** Phylogeny of the *G. raimondii* DREB (dehydration responsive element binding gene) and ERF (ethylene responsive factor) subfamily proteins based on their conserved domain sequences. Different subgroups of DREB and ERF subfamily proteins are highlighted in different colors

In order to appreciate the phylogenetic relationships of the *AP2/EREBP* genes in *G. raimondii*, we employed MEGA v5.2 software to construct an unrooted phylogenetic tree of AP2/EREBPs from *G. raimondii* and *A. thaliana* (Additional file 5: Figure S3). The phylogenetic tree clearly showed that the remaining 265 genes were clustered into four subfamilies, the RAV, AP2, DREB and ERF clades, comprising 11, 32, 80 and 142 proteins, respectively (Fig. 1). This was consistent with the above classification that was based on domain compositions and the number of AP2 domains.

The classifications of the AP2/EREBP genes were used to infer the phylogenetic history of the DREB/ERF genes and AP2 and RAV genes, respectively (Fig. 1). As shown in the phylogenic tree, the 32 AP2 genes and the 11 RAV genes were divided into two subfamilies based on their sequence similarities and the number of AP2/ERF domains in the encoded proteins (Fig. 1a). The remaining 222 genes were classified into two subfamilies: the DREB (80 genes) and ERF (142 genes) subfamilies (Fig. 1b). There were two conserved amino acids distinguished between the DREB and ERF genes (Additional file 4: Figure S2). The 19th glutamic acid (E19), and, in particular, the 14th valine (V14) were conserved in the DREB proteins, whereas alanine (A14) and aspartic acid (D19) were conserved in the ERF proteins (Additional file 4: Figure S2). The DREBs specifically bound to six nucleotides (A/GCCGAC) of DRE, while the ERFs were found to be involved in ethylene-responses and specifically bound to the GCC box (AGCCGCC) [33]; indicating the functional divergence of the DREB and ERF subfamilies in plants.

Moreover, the DREB subfamily was further divided into six groups (A1 to A6) as in Arabidopsis [11], of which the A4 group was the largest and the A3 group was the smallest with only one member (Gorai.011G291900) (Fig. 1b). Likewise, the ERF subfamily was classified into seven groups as in rice [15] and *Brachypodium Distachyon* [18]. The group B3 contained 63 (44% of the 142 ERFs) members and was the largest (Fig. 1b). Interestingly, the number of ERF subfamily members was almost double that of the DREB subfamily members. This indicated that each AP2/EREBP subfamily evolved to have species-specific characteristics and the ERF subfamily may have experienced fast gene expansion.

The ERF subfamily members included more than half of the AP2/DREBP superfamily members, including five genes (*GrERF23*, *GrERF83*, *GrERF82*, *GrERF136* and *GrERF55*) that had not been reported before and could not be allocated into any existing group (B1 to B6), so were classified into a new group, B7, as in rice and *Brachypodium Distachyon* [15, 18]. These genes may have new functions related to human selection pressure during cotton domestication.

### Chromosomal locations of *GrAP2/EREBP* genes

**Fig. 2:**
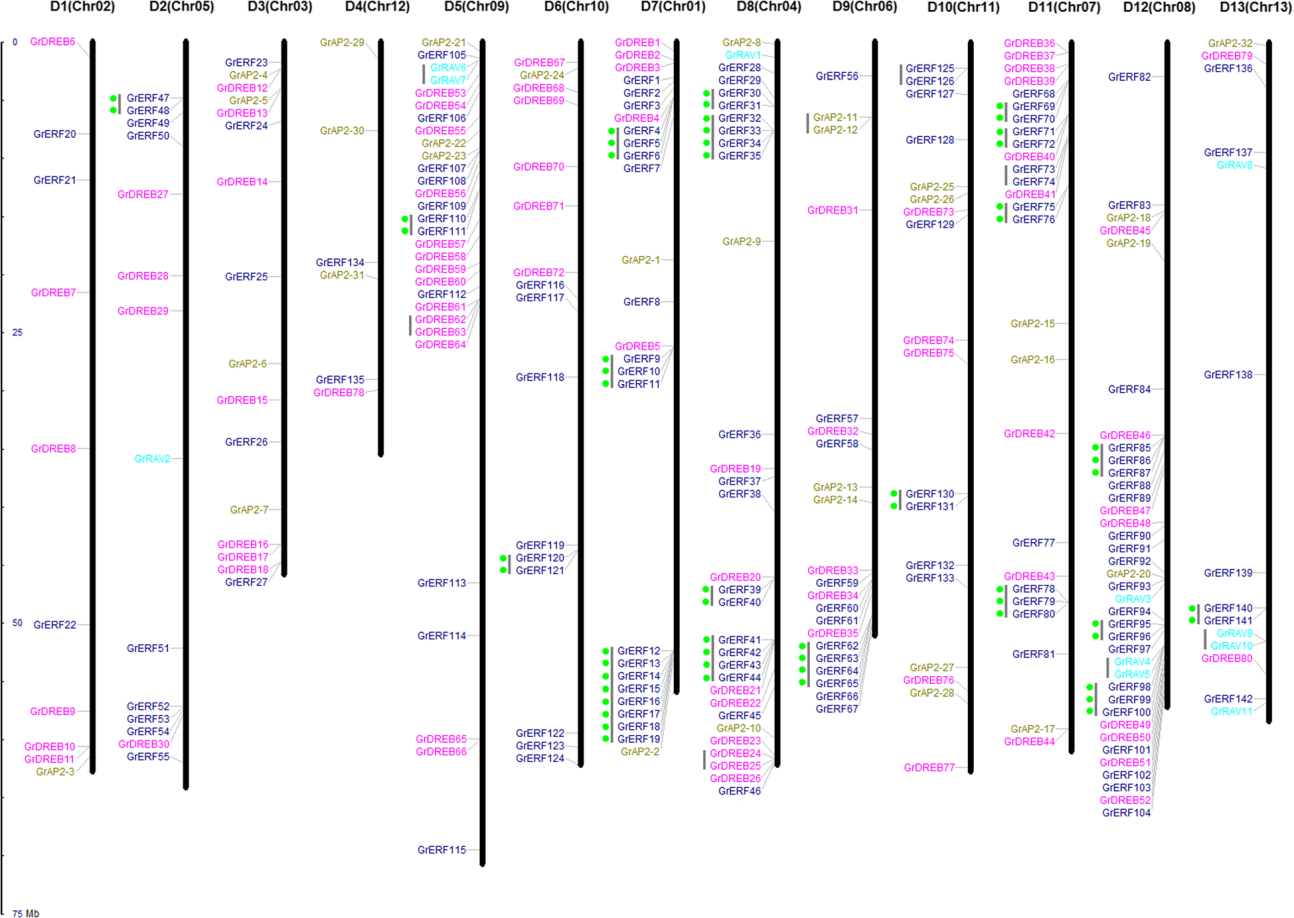
Chromosomal distributions of the *G. raimondii AP2/EREBP* genes. The chromosome numbers were consistent with those in the interspecific genetic map (D1 to D13) of allotetraploid cultivated cotton species and the scaffolds (Chr.1 to Chr.13) of the *G. raimondii* genomic data [43]. The nomenclature of the AP2/EREBP genes was based on the order of the chromosomes in *G. raimondii. Grey lines* indicate tandem duplicated genes. Genes are color coded according to their subfamily

To examine the genomic distribution of *AP2/EREBP* genes on cotton chromosomes, we identified their positions. The 265 cotton *AP2/EREBP* genes were scattered unevenly over the 13 chromosomes, and were distributed individually or in clusters (Fig. 2). Chromosome D12 had the largest number (38) of *AP2/EREBP* genes, while chromosome D4 had the smallest, with only six members (*GrAP2-29*, *GrAP2-30*, *GrAP2-31*, *GrDREB78*, *GrERF134* and *GrERF135*). Interestingly, many genes were distributed in clusters, especially at the telomeric ends of chromosomes 5, 7, 8, 9, 11 and 12 (Fig. 2). Such uneven distribution of these genes provided a clue to their evolution.

Some genes distributed in clusters were tandem duplicated genes. We analyzed possible tandem duplication events in the *G. raimondii* AP2/EREBP family and found that 73 *AP2/EREBP* genes belonging to 28 gene clusters were involved in tandem duplication (Fig. 2). The number of *GrAP2/EREBP* duplicated genes arranged in tandem repeats varied from two to eight, and the largest gene cluster consisted of eight tandem duplicated genes (*GrERF12-19*) on chromosome D7. There were 57 tandem duplicated genes belonging to group B3 of the ERF subfamily (Fig. 2 and Additional file 6: Table S3). Additionally, there were six genes (three pairs of RAVs) originating from tandem duplication events. This is a large proportion for a relatively small subfamily.

### The exon/intron organizations of *GrAP2/EREBP* genes are relatively simple

To obtain a deep insight into the gene structures of the cotton AP2/EREBP genes, their intron and exon structures were analyzed. We found that the *AP2/EREBPs* had a relatively simple gene structure, with the exception of the AP2 subfamily members (Additional file 7: Figure S4). All members of the AP2 subfamily had five to nine introns, while the majority of members of the RAV, DREB and ERF subfamilies had no introns and relatively simple structures. All members of the DREB and RAV subfamilies had no introns, with the exception of *GrDREB7*, *GrDREB13*, *GrDREB24*, *GrDREB28*, *GrDREB54*, *GrDREB57*, *GrDREB67*, *GrDREB74* and *GrRAV5*, which had one intron, and *GrDREB15* and *GrRAVA4*, which had two introns. In the ERF subfamily, all members of the groups B2 and B4 had one intron and most members of the other B groups had no introns or one intron, with the exception of *GrERF58* and *GrERF105*, which had two introns. In addition, the 63 members of the largest group, B3, had no introns, with the exception of *GrERF13*, *GrERF16*, *GrERF39*, *GrERF69*, *GrERF78*, *GrERF99* and *GrERF134*, which had only one intron each (Additional file 7: Figure S4). Genes originating from tandem duplications were clustered together in the phylogenetic tree and shared similar structural organizations (Additional file 7: Figure S4).

### AP2/EREBP superfamily expansion in *Gossypium*

**Fig. 3:**
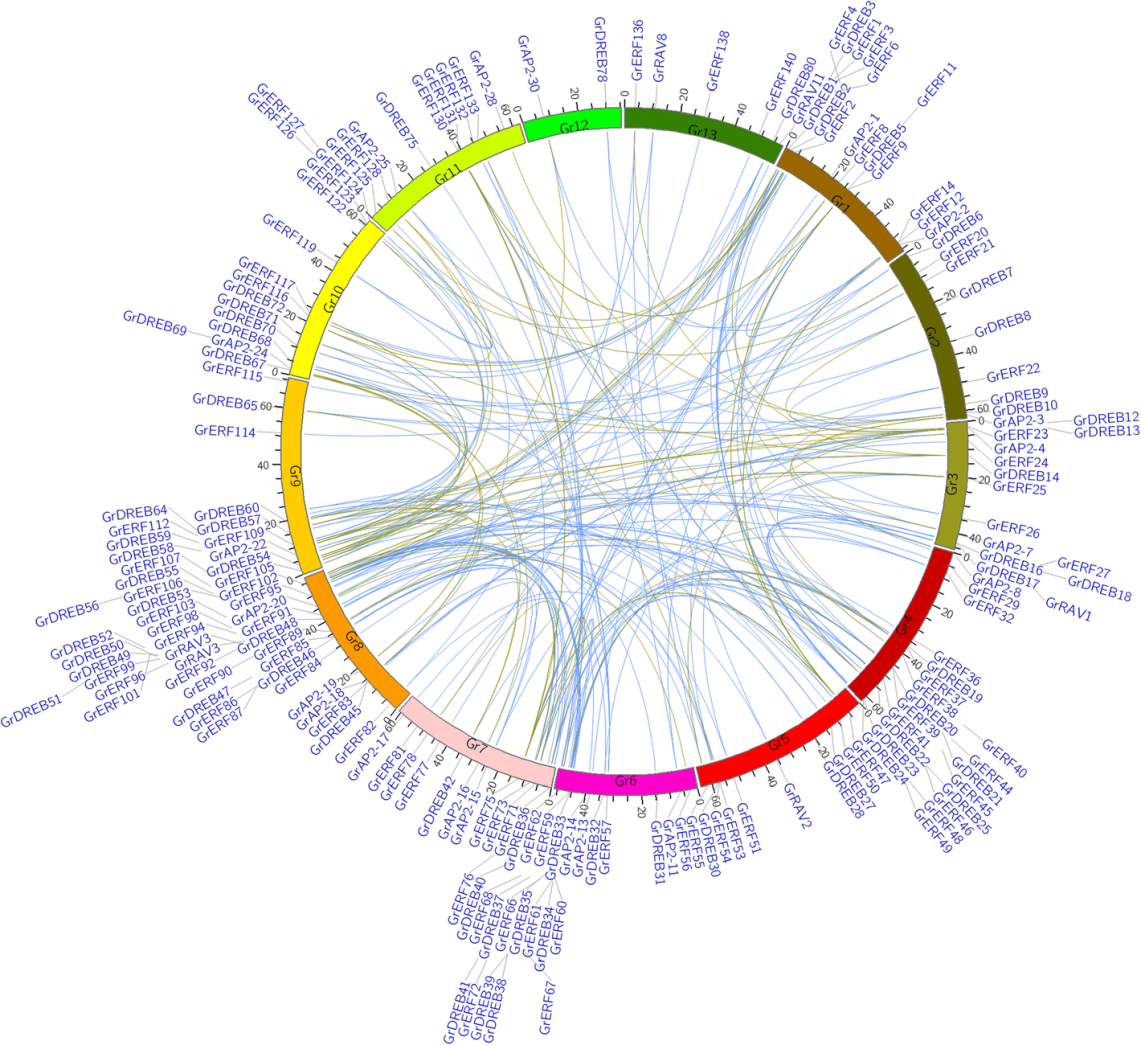
Synteny relationships between *AP2/EREBP* genes in *G. raimondii. Blue lines* show duplications with Ks >1.5 and *green lines* show duplications with Ks <1.5

Tandem and segmental duplications are known to be major forces for expansion of gene families in plants. Studies showed it is important of gene family expansion of plant tandem duplicates in the adaptive response to environmental stimuli [45, 46]. Large-scale duplication events are predicted to have occurred during *Gossypium* evolution [43]. We analyzed the possible tandem and segmental duplication events in the *G. raimondii* AP2/EREBP family and found 73 genes in 28 tandem gene clusters distributed unevenly on ten cotton chromosomes (Fig. 2), as well as 221 segmental duplicated gene pairs in 185 blocks, which were found throughout the genome (Fig. 3).

There were 57 tandem duplicated genes belonging to group B3 of the ERF subfamily and they contributed 82.6% of all the duplicated genes (Fig. 2 and Additional file 6: Table S3). The largest gene cluster consisted of eight tandem duplicated genes on chromosome D7 (Fig. 2). Tandem duplications made a great contribution to the expansion of the group B3 of the ERF subfamily.

To further investigate the AP2/EREBP gene family expansion pattern in *G. raimondii*, we download the syntenic data of *G. raimondii* from the Plant Genome Duplication Database (PGDD) and analyzed the gene duplication pattern of AP2/EREBPs from different phylogenetic subfamilies. AP2/EREBPs with syntenic relationships were detected in all subfamilies, indicating extensive segmental duplications throughout the genome (Fig. 3). In total, there were 221 pairs of genes with syntenic relationships in 185 syntenic blocks. Of them, there were 83 pairs of DREB, 112 pairs of ERF, 22 pairs of AP2 and four pairs of RAV subfamily genes (Fig. 3). In summary, *Gossypium* lineage segmental duplication events contributed to the expansion of all four subfamilies. However, most of the tandem duplicated genes (82.6%) belonged to group B3 of the ERF subfamily and contributed to its expansion.

**Fig. 4:**
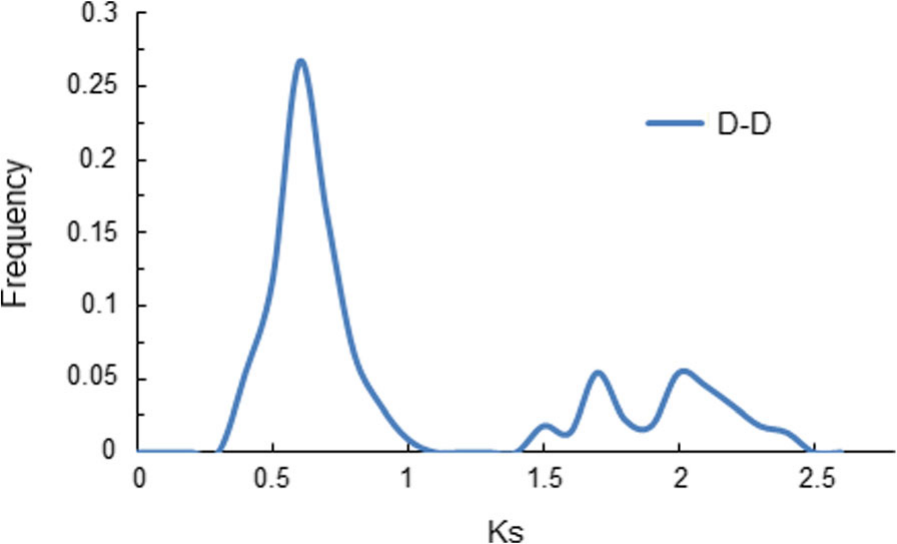
Frequency distribution of Ks values of segmental duplicated gene pairs in *G. raimondii.* The x axis denotes the Ks value. The y axis denotes the relative frequency

Furthermore, we analyzed the substitution per synonymous site (*Ks*) values of 221 segmental gene pairs in *G. raimondii* and observed two peaks at *Ks* values of 0.5–0.8 and 1.5–2.3 (Fig. 4 and Additional file 8: Table S4). The first peak appeared at approximately 60 Mya, corresponding to the whole-genome duplication (WGD) event that was previously proposed in the *Gossypium* lineage [43, 47]. The second peak appeared at about 130.8 Mya, corresponding to the paleohexaploidization event shared by the eudicots [43, 48–50]. The *Ks* values for each pair of genes within a syntenic block were used to interpret duplication events (Additional file 8: Table S4). The 64 paralog gene pairs with *Ks* values ranged from 1.50 to 2.45, may be derived from the ancient hexaploidization event about 130.8 Mya [46, 50]. The remaining 157 paralog gene pairs, which had *Ks* values ranged from 0.45 to 1.08, were likely to have originated from the *Gossypium* lineage duplication events [43, 47]. Interestingly, we found that no RAV subfamily genes were retained after the ancient hexaploidization event, while all four pairs of RAVs (*GrRAV1 & GrRAV11*, *GrRAV2 & GrRAV8*, *GrRAV2 & GrRAV3* and *GrRAV3 & GrRAV8*) with syntenic relationships originated from the recent *Gossypium* lineage duplication events.

### Synteny comparison of AP2/EREBP subfamily to cacao and grape

A genomic analysis of the synteny of the AP2/EREBP gene family across *Theobroma cacao* and *Vitis vinifera* could provide insights to its expansion and evolution. We comparatively analyzed the synteny across *G. raimondii*, *T. cacao* and *V. vinifera* (Additional file 9: Figure S5). There are 312 syntenic gene pairs that were syntenic between *G. raimondii* and *T. cacao*, while there are 127 pairs between *G. raimondii* and *V. vinifera* (Additional file 9: Figure S5). The greater number of segmental duplicated paralogous gene pairs between *G. raimondii* and *T. cacao* indicates the closer relationship between *G. raimondii* and *T. cacao* than that between *G. raimondii* and *V. vinifera*. These results were consistent with the evolutionary relationships already identified between these species [43].

In this study, we identified a total of 269 *AP2/EREBP* genes in the diploid cotton *G. raimondii*, 102 in grape and 123 in cacao. These 269 AP2/EREBPs represented 0.72% of the whole genome of *G. raimondii*, a ratio much higher than the 0.39% in grape [24], 0.55% in Arabidopsis [11] and 0.42% in cacao, suggesting that the expansion of the AP2/EREBPs in cotton was greater than that of in other dicot plants. This expansion may have benefited from the *Gossypium* lineage WGD events [43, 47]. Furthermore, we compared the proportion of genes in each of the subfamilies in *G. raimondii*, cacao and grape and found that the number of genes in the DREB, ERF and RAV subfamilies increasing during the divergence of the species. However, the number of AP2 subfamily genes decreased gradually, while the number of outsider genes remained unchanged (Additional file 10: Figure S6). These results indicate the expansion of the AP2/EREBP superfamily was mainly due to the expansion of DREB and ERF subfamilies by segmental or tandem duplications, and this expansion may have enhanced the wide adaptability of cotton.

### Spatio-temporal expression profiles of *GhAP2/EREBP* genes in *G. hirsutum*

**Fig. 5:**
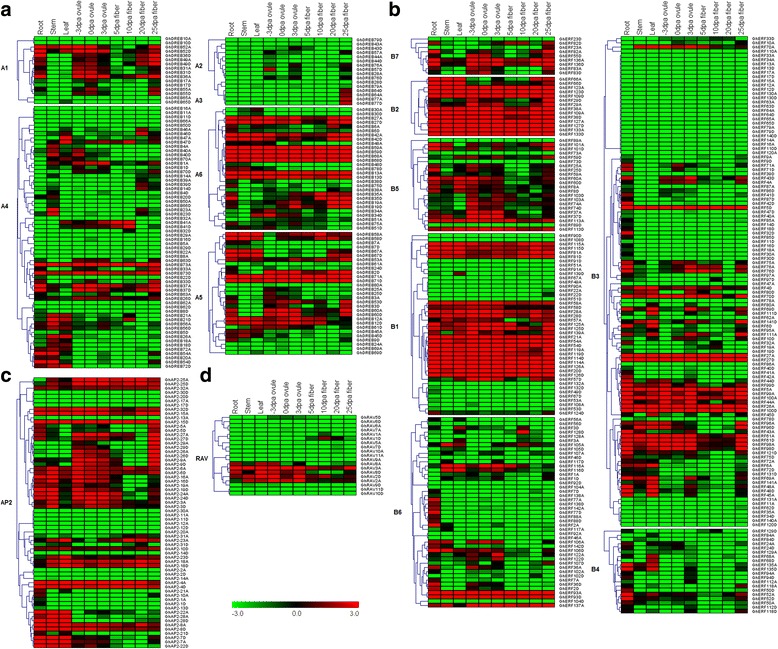
Expression profiles of *GhAP2/EREBP* genes in various tissues. Ten tissues comprising roots, stems, leaves, −3dpa ovules, 0dpa ovules, 3dpa ovules, 5dpa fibers, 10dpa fibers, 20dpa fibers and 25dpa fibers were investigated. Expression profiles (in log2 based values) of the *GhDREB* (**a**), *GhERF* (**b**), *GhAP2* (**c**) and *GhRAV* (**d**) genes in three tissues. Scale bars represent log2 of the RPKM values

*AP2/EREBP* genes play essential roles in plant growth and development including root initiation in rice [51], ovule and sepal development in Arabidopsis [22, 52], fruit development and ripening process in tomato [23] and grapevine growth [24]. We used our high-throughput sequencing data from *G. hirsutum* acc. TM-1 [42] to investigate the expression profiles of *AP2/EREBP* family genes in various *G. hirsutum* tissues, including roots, stems, leaves, −3 days post anthesis (dpa), 0 dpa and 3 dpa ovules and 5 dpa, 10 dpa, 20 dpa and 25 dpa fibers. There were 504 *AP2/EREBP* genes expressed at least in one tested tissue. To further elucidate the transcription patterns of *GhAP2/EREBP* genes, their expression patterns were clustered across each subfamily and different groups. In general, different subfamilies showed different expression patterns (Fig. 5), suggesting the functional divergence of different subfamilies and groups of *GhAP2/EREBP* members.

The *DREB* genes, showed various expression patterns and functional divergence in vegetative and reproductive organs (Fig. 5a). Five genes in group A1 were highly expressed in roots, −3 dpa ovules and 0 dpa ovules, as well as in 25 dpa fibers. Two genes (*GhDREB52A/D*) were expressed in all of the ten tissues investigated, except 5 pda and 10 dpa fibers. Three genes were only expressed in one or two tissues. In addition, two pairs of homoeologous genes (*GhDREB52A/D* and *GhDREB55A/D*) showed obvious subgenome expression bias in different tissues (Fig. 5a), revealing the functional divergence of homoeologous gene pairs in allotetraploid cotton. Only four genes in the groups A2 and A3 were highly expressed in only one tissue. And in the groups A5 and A6, more genes were constitutively expression in all tissues. The A4 group contained the greatest number of members but about half of them showed very little expression (Fig. 5a).

The ERF members represented 52.8% (142 genes) of the whole AP2/EREBP family and had the greatest number of expression profiles in various tissues (Fig. 5b). Almost all genes in group B2, and half of the members of group B1, showed constitutive expression, but the group B4 members were only detected in roots or leaves and had lower expression levels. In contrast, members of the groups B3, B5, B6 and B7 showed relatively diverse expression patterns (Fig. 5b). For example, members of group B3, the largest group in the AP2/EREBP family, showed very little expression in any tissue or showed biased expression of one of the homoeologous gene. As a newly classified group [15], members of the group B7 showed very high or very low expression levels, and there was a great divergence in function between those in A- and D-subgenomes (Fig. 5b). The ERF subfamily contributed greatly to the expansion of the AP2/EREBP family, and the genes in this group showed various expression patterns indicating functional divergence during the long period of evolution.

The AP2 genes have been reported to play essential roles in plant growth and development [14, 22–24, 51, 52]. Here, we found 15 genes that were constitutively expressed, 17 genes expressed in the early ovule stage, and ten genes that were mainly expressed in 25 dpa fibers (Fig. 5c). At present, little is known about the role of RAV genes in plant growth and development. Reports have showed the RAV genes may mediate plant responses to auxin, which is involved in plant development [27, 53, 54]. Here, we found only five genes that were expressed mainly in vegetative organs and only one that was specific to 10 dpa fibers. Of them, the *GhRAV1D* gene was most highly expressed in 10 dpa fibers and may therefore play a role in fiber elongation (Fig. 5d).

### Many *GhDREB/ERF* genes were induced expression under abiotic stresses

To investigate the potential functions of the *GhAP2/EREBP* genes under various environmental stresses, we used RNA-seq data [42] to detect their expression levels under cold, heat and drought conditions. A total of 151 *GhDREB* and *GhERF* genes, including 60 *GhDREBs* and 91 *GhERFs*, which were induced by stress treatments were subjected to expression analysis under these stresses. Of these, the expression of 132 genes was induced by cold, 94 genes by heat and 63 genes by drought (Additional file 11: Figure S7). Therefore, the greatest number of these genes was induced by cold and the lowest by drought.

**Fig. 6:**
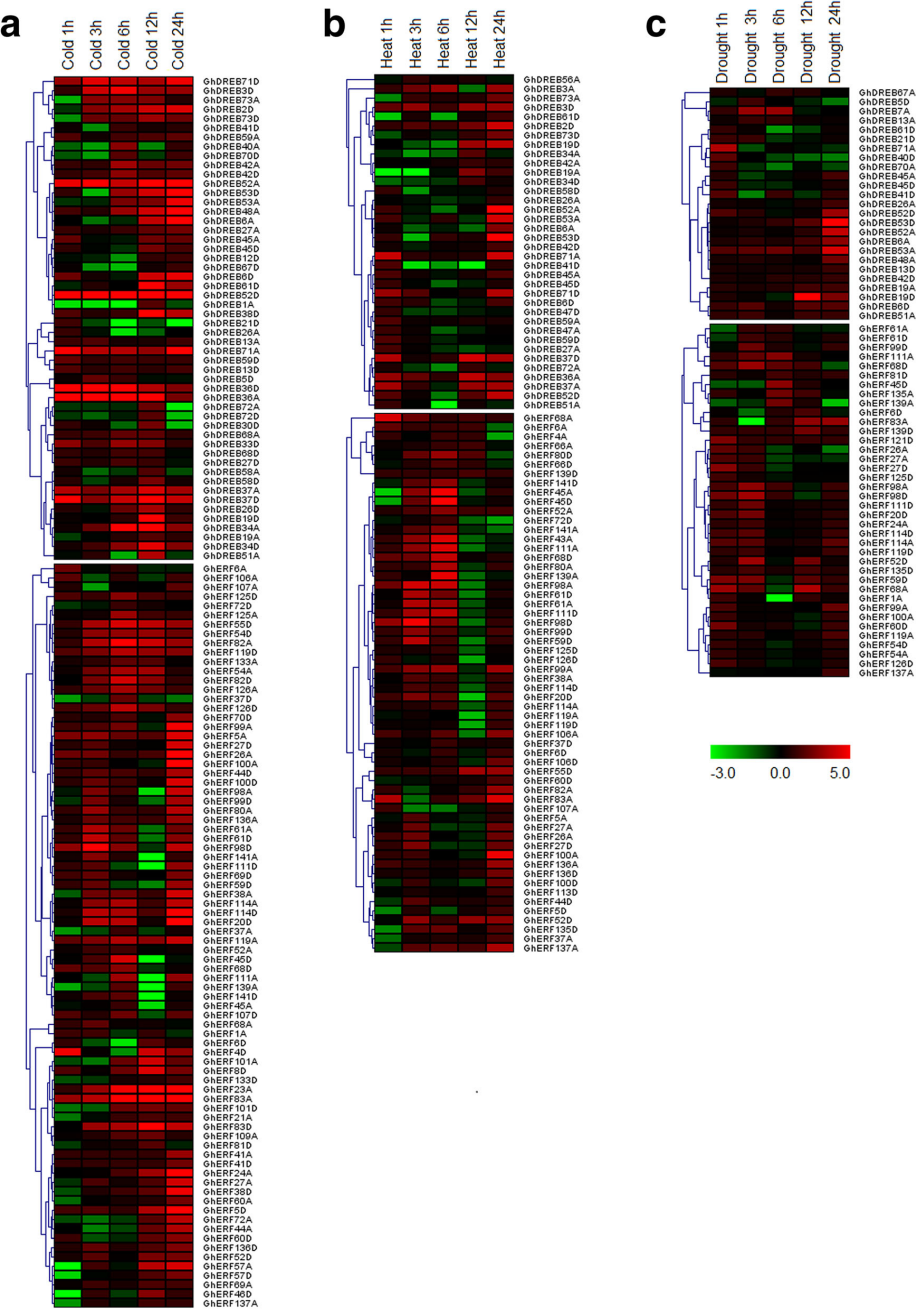
Expression patterns of *GhDREB/ERF* genes under various stresses. Expression profiles (in log2 based fold change) of *GhDREB* and *GhERF* genes under different abiotic stress conditions: including **a** cold, **b** heat and **c** drought. *Scale bars* represent log2 of the RPKM values

The *GhDREB* subfamily genes could be classified into two subgroups based on their expression profiles in cold conditions. In the first group, the peak of *GhDREB* genes expression occurred as late as 12 or 24 h after stress treatment, while the other group of *GhDREB* genes had an expression peak 24 h after the treatment. In addition, 11 of these genes showed constitutive expression (Fig. 6a). In some of the homoeologous gene pairs, only one gene was induced, while the other was not detected, indicating that it may lost its function after the formation of allotetraploid cotton. There were three main expression patterns of the *GhERF* genes. In the first group, they responded rapidly in the early stage of induction and reached a peak at 6 h (for example *GhERF125A* and *GhERF82D*) (Fig. 6a); in the second group, they were induced in the early stage, then their expression declined, before increasing to reach a second peak; and in the third group, they were induced relatively slowly, and reached an expression peak at 24 h or more after stress treatment (Fig. 6a).

The number of genes induced by heat treatment were lower than that in cold and drought treatments, and they showed different expression levels and different durations of expression. With the Exception of a small number of *GhDREB* genes, which were expressed continuously, most of the other genes had two expression peaks at 1 h and 24 h after treatment (Fig. 6b). The genes induced by heat were divided into two groups based on their expression profiles; those that reached a peak at 6 h and continued being highly expressed until at least 24 h after treatment, and those that were mainly expressed at 3 h and 24 h after treatment (Fig. 6c). *GhDERB* genes are known to mediate changes in gene expression in drought and low temperature conditions [55]. Here, we found that most of the *GhDREB* genes were rapidly induced during early stages of drought treatment. The expression of some of *GhDREB* genes then decreased, while some others increased over time (Fig. 6c). These results suggest that different *GhDREB* genes play different roles in drought responses. Some of the *GhERF* genes were also induced by drought at early or late stages, this showed various expression patterns of *GhERF* genes (Fig. 6c). Multiple stresses induced expressions were found between *GhDREB* and *GhERF* genes under different stress treatments, indicating that the *GhDREB* and *GhERF* genes may be involved in a crosstalk between signal transduction pathways in response to different abiotic stresses, or that some functions of different genes are complementary.

**Fig. 7:**
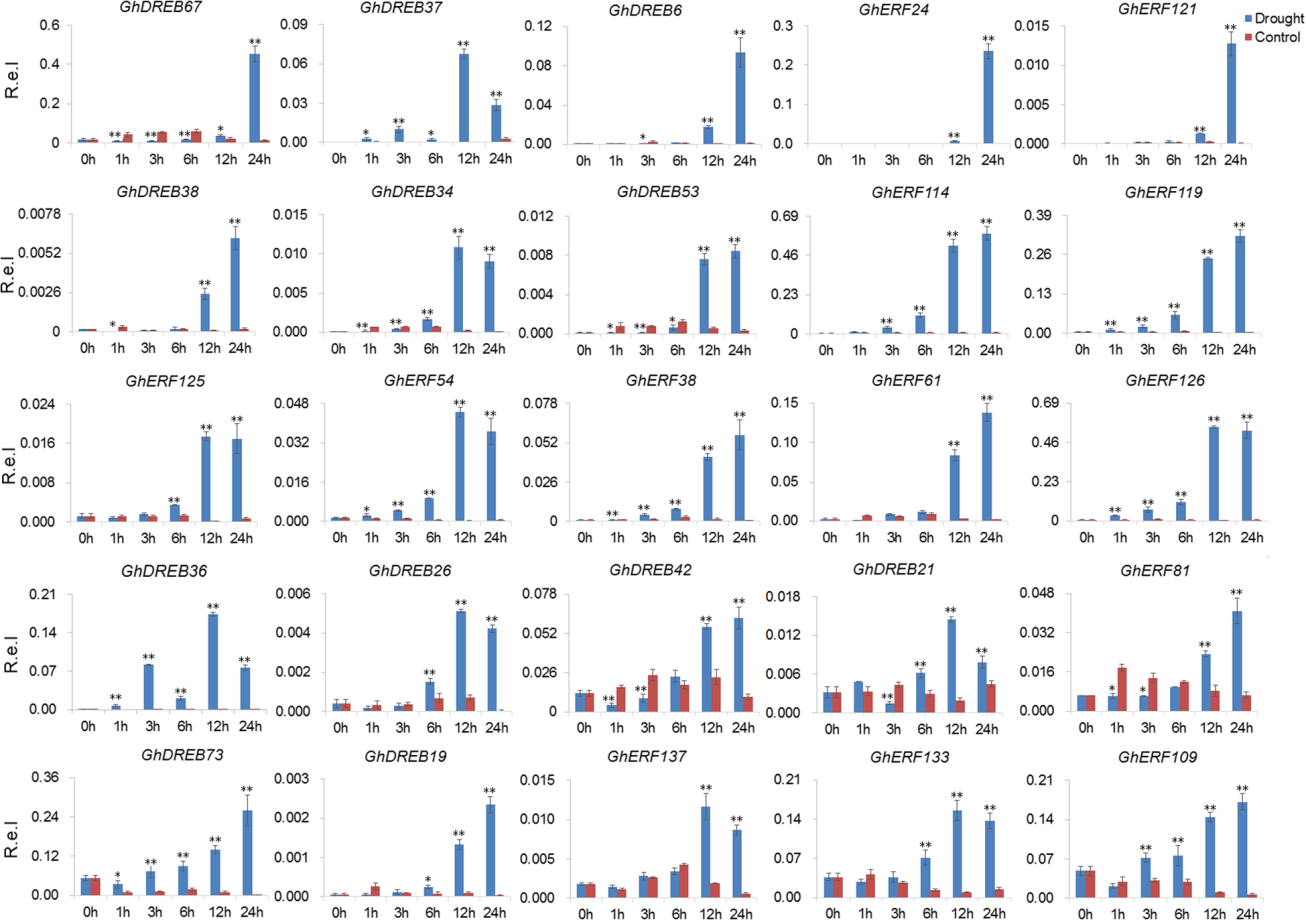
Validation the expression of the selected *GhDREB/ERF* genes in response to cold using qRT-PCR. The mean expression values were calculated from three independent replicates. 0, 1, 3, 6, 12 and 24 h indicate the number of hours after treatment. Mean values and standard errors were calculated from three replicates. The *asterisk* and *double asterisks* represent significant differences at the levels of 0.05 and 0.01, respectively. R.e.l indicates Relative expression level

**Fig. 8:**
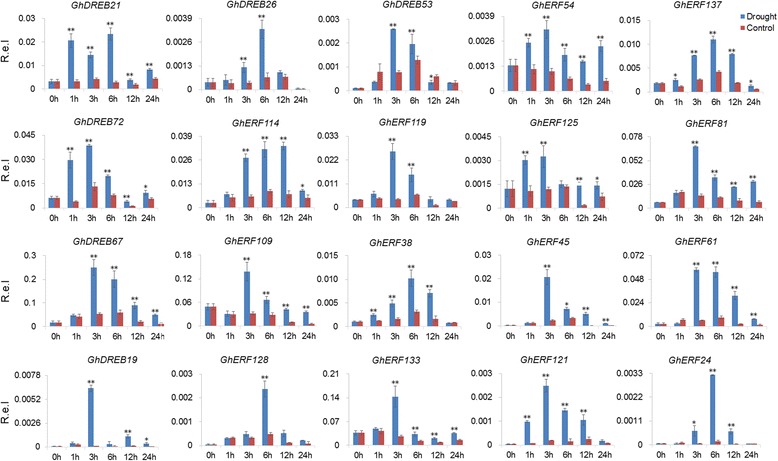
Validation the expression of the selected *GhDREB/ERF* genes inresponse to drought using qRT-PCR. The mean expression values were calculated from three independent replicates. 0, 1, 3, 6, 12 and 24 h indicate the number of hours after treatment. Mean values and standard errors were calculated from three replicates. The *asterisk* and *double asterisks* represent significant differences at the levels of 0.05 and 0.01, respectively. R.e.l indicates Relative expression level

We also examined the expression of 28 selected genes (13 *GhDREBs* and 15 *GhERFs*) using quantitative reverse transcription PCR (qRT-PCR) under cold and drought stress treatments. 17 genes were induced by both cold and drought stresses. All of the 28 selected genes were significantly induced by stress at one or more time point/s (Figs. 7 and 8), and this was consistent with the RNA-Seq data. Overall, the gene response was slower in cold conditions than in drought conditions. Expression levels gradually increased over time in low temperature conditions and reached a peak at 12 h or 24 h (Fig. 7). In contrast, genes responded immediately to drought, with expression levels peaking 1 or 3 h after treated (Fig. 8). These results indicate that the expression patterns of *GhDREB/ERF* genes under cold damage might improve the environmental adaptability of cotton to high latitude regions with relatively lower temperatures.

### Functional divergence of *GhAP2/EREBP* genes

Gene duplication is a major force of genetic novelty and can lead to evolutionary innovation. Three possible fates of duplicated genes have been suggested as non-functionalization, sub-functionalization and neo-functionalization [56]. Gene expression patterns analysis may provide a lot of information for studying the functional divergence and evolution of homoeologous genes. Using RNA-seq data [42], the expression patterns of homoeologous gene pairs in cotton were compared to gain insights into their functional divergence during evolution.

Most of the homoeologous gene pairs showed no expression bias between homoeologs. These genes may retain their original function based on the gene dosage. But some homoeologous gene pairs with one member lost or silenced showed non-functionalization in allotetraploid cotton. For example, *GhDREB2A*, *GhERF55A* and *GhRAV3D* had function in specific organs, while their homoeologs was silenced (Additional file 12: Figure S8a). Possible explanations for this are many and varied, but include the preservation of appropriate gene dosage and the requirements imposed by interacting and divergent regulatory hierarchies [57, 58].

There were also some gene pairs that had partitioned aggregate ancestral functions (sub-functionalization), for example, it is showed half gene dosage of each member of the *GhDREB12*, *GhDREB77* and *GhERF81* homoeologous gene pairs (Additional file 12: Figure S8b). However, the *GhAP2-31* and *GhDREB26* homoeolog pairs showed expression level dominance in different tissues. The *GhAP2-31A* and *GhDREB26D* genes had lower expression levels in vegetative organs, such as roots, stems and leaves than those of *GhAP2-31D* and *GhDREB26A*, but had higher expression levels in reproductive organs including ovules and fibers (Additional file 12: Figure S8c). These results indicate non-functionalization and sub-functionalization of homoeologous gene pairs during cotton evolution.

We also noticed that one member of the homoeologous gene pairs were differentially induced by different stress may indicate neo-functionalization of one member. For example, the homoeologous gene pairs of *GhDREB1*, *GhDREB7*, *GhDREB12*, *GhDREB33*, *GhDREB40*, *GhDREB56*, *GhERF70*, *GhERF109* and *GhERF113* were both expressed in various tissues but only one member such as *GhDREB1A*, *GhDREB12D*, *GhDREB33D*, *GhERF70D* and *GhERF109A* were induced by cold stress, *GhDREB56A* and *GhERF113D* were induced by heat stress, *GhDREB40D* was induced by drought stress and *GhDREB7A* was induced by drought, heat and cold stresses (Figs. 5 and 6). One member of these homoeologous gene pairs may gain new function (neo-functionalization) in cotton stress tolerance.

Additionally, we analyzed the expression patterns of 111 tandem duplicated genes from 41 tandem clusters and detected no transcripts for almost half of them in select tissues (Additional file 13: Figure S9). No transcripts were detected for either member of some tandem duplicated pairs, for example, *GhAP2-11A* and *GhAP2-12A*, *GhAP2-11D* and *GhAP2-12D*, *GhERF64A* and *GhERF65A*, *GhRAV6A* and *GhRAV7A*, *GhRAV9A* and *GhRAV10A*, *GhRAV6D* and *GhRAV7D*, *GhERF62A* and *GhERF63A*, *GhERF39D* and *GhERF40D*, *GhERF64D* and *GhERF65D*, *GhRAV10D* and *GhRAV9D* and *GhERF62D* and *GhERF63D*. In contrast, there were some tandem duplicated clusters in which all members were expressed, for example, *GhERF41A*, *GhERF42A*, *GhERF43A* and *GhERF44A*, *GhERF125A* and *GhERF126A*, *GhERF69A* and *GhERF70A*, *GhERF71A* and *GhERF72A*, *GhERF75A* and *GhERF76A*, *GhERF98A*, *GhERF99A* and *GhERF100A*, *GhERF4A*, *GhERF5A* and *GhERF6A*, *GhERF41D*, *GhERF42D*, *GhERF43D* and *GhERF44D*, *GhERF125D* and *GhERF126D*, *GhERF69D* and *GhERF70D*, *GhERF71D* and *GhERF72D*, *GhERF75D* and *GhERF76D*, *GhERF98D*, *GhERF99D* and *GhERF100D*, *GhERF4D* and *GhERF5D* and *GhERF6D*. Transcripts of only some members of the remaining tandem duplicated gene clusters were detected (Additional file 13: Figure S9). This indicates that some tandem duplicated genes in the young allotetraploid cotton are functionally redundant and might have lost or gain functions during the long domestication process.

In short, the expression analysis of duplicated genes in the A- and D-subgenomes (homoeologous genes) and tandem duplicated genes revealed their functional roles for non-functionalization, sub-functionalization and neo-functionalization during the expansion of the AP2/EREBP superfamily *in G. hirsutum*. In other words, the functions of the superfamily genes were expanded and enhanced by gene duplication and genome doubling.

## Discussion

### EREBP genes expanded obviously in cotton

WGD or polyploidy, which results in massive silencing and elimination of duplicated genes, has long been recognized as a significant force in plant evolution [59]. In this study, we identified 269 *AP2/EREBP* genes in *G. raimondii*, representing 0.72% of the annotated proteins in this species. The higher ratio (0.72%) suggested expansion of the AP2/EREBP family in *Gossypium*. The ratios of RAV and AP2 subfamily genes were nearly the same as that in Arabidopsis [11] and poplar [12], showing a strong conservation of these subfamilies and suggesting that the RAV and AP2 genes in all of these plants share a common ancestor prior to the separation of *raimondii* from the other plants. However, the number of DREB and, in particular, the ERF genes in cotton changes significantly, indicating that they have independently expanded after the cotton differentiation from other species. The group B3 contained the greatest number of members (63 genes) of all the gene groups and expanded mainly form tandem duplication. This indicates the B3 group of the ERF subfamily may experience special functional divergence during cotton evolution. Of the 73 tandem duplicated genes in *G. raimondii*, 57 members belonged to group B3 of the ERF subfamily. The remaining 16 genes comprised three pairs of RAVs (*RAV4 & RAV5*, *RAV6 & RAV7*, *RAV9 & RAV10*,), one pair of AP2s (*AP2-11 & AP2-12*) and two pairs of ERFs (*ERF73 & ERF74*) (Fig. 2). Studies showed it is important of gene family expansion of plant tandem duplicates in the adaptive response to environmental stimuli [45, 46]. The expansion of the ERFBP gene family is an important force for functional divergence to stress response, especially of the B3 group of the ERF subfamily. These genes provided clues to the evolution of tandem duplicated genes and stress tolerance improvement of cotton.

### Simple gene structures may reveal high conserved functions

The domain architecture and intron/exon structure of the *AP2/EREBP* genes in *G. raimondii* were relatively simple and highly conserved within each subfamily (Additional file 4: Figure S2 and Additional file 7: Figure S4). The DREB and ERF subfamily genes were mainly classified based on two conserved amino acids, the 14th valine (V14) and the 19th glutamic acid (E19) of the DREB proteins, which were alanine (A14) and aspartic acid (D19) of the ERF proteins (Additional file 4: Figure S2). The valine (V14) was present in all the DREB members but the glutamic acid (E19) was not, indicating functional divergence of the A groups. For example, *GhDREB76* (A19) and *GhDREB79* (V19) of group A2 showed different expression patterns in various tissues (Fig. 5a). In addition, in the A5 and A6 groups, there were only six *DREB* genes that contained E19; the others contained A19 or L19 (Additional file 4: Figure S2). This is consistent with the expression patterns of the two groups with more members than the other groups, which were highly expressed in all selected tissues (Additional file 4: Figure S2a). The results imply high levels of conservation of V14 in the DREB genes, but divergence in the 19th amino acid contributed to functional variation. In general, there were few differences observed between the ERF members, with the exception of group B6, in which one or two conserved amino acids changed (Additional file 4: Figure S2b). Interestingly, the expression patterns of group B6 members also had distinctive characteristics. In detail, most of the genes in group B6 were specifically expressed in one or several organs (Fig. 5b). This indicates that functional divergence between DREB and ERF genes occurred via change in critical amino acids during cotton evolution and provides excellent candidate genes for researching cotton developmental regulation.

### Excellent candidates for cotton improvement

In this paper, we identified some important candidate genes which were highly or specific expressed in some tissues, such as *GhDREB77* in fiber maturation, *GhDREB2* in −3 dpa ovules involved in fiber initiation (Additional file 12: Figure S8), *GhERF77* and *GhERF88* in root development, *GhERF139D* in leaf development and *GhRAV1* in fiber elongation (Fig. 5). The genes involved in different stresses, tissues and developmental stages provide fine candidates for cotton improvement. Zhou et al. [60] identified *GhERF12* in *G. barbadense*, which is involved in cotton seedling growth and development and induced by 1-aminocyclopropane-1carboxylic acid (ACC) and indole-3-acetic acid (IAA). It is homologous to *GhERF76*, which was identified in this study in cotton roots, ovules and fibers and may play a role in cotton root and fiber development.

Studies have shown that *DREB* and *ERF* genes are responsive to abiotic stress, so we investigated the expression profiles of *DREB* and *ERF* genes in *G. hirsutum* under stress treatments using RNA-seq data and qRT-PCR validation. As expected, a large number of *DREB* and *ERF* genes were induced by cold, heat and drought (Figs. 6, 7 and 8). Several *DREB* and *ERF* genes were induced by multiple stresses, revealing crosstalk between multiple environmental stimuli. This may provide clues for the studying of crosstalk in different stress conditions. In addition, overexpression of *ERF* genes resulted in improved tolerance to drought and salt in Egyptian clover [61], osmotic stress in tobacco [62], and cold, drought and heat responses in Arabidopsis [63]. These genes in cotton were induced by cold, heat or drought (Fig. 6), suggesting widespread and conserved tolerance to stress via *DREB* and *ERF* genes in different plant species. Of the 28 selected genes, 17 *GhDREB* and *GhERF* genes were induced by both cold and drought (Fig. 7). Genome wide analysis of *AP2/EREBP* genes will provided candidates for cotton stress tolerance improvement.

### The fates of cotton homoeologous genes

Duplicated or homoeologous genes in plants experienced different fates, including non-functionalization, neo-functionalization or partitioned aggregate ancestral functions (sub-functionalization) [64]. The expression profiles of the tissues examined in this study provide examples of the functional divergence of *AP2/EREBP* genes. As shown in Additional file 12: Figure S8, the expression levels of members of the *AP2/EREBP* family in *G. hirsutum* differed in the different tissues. Additionally, almost half of the tandem duplicated genes were not expressed (Additional file 13: Figure S9), indicating functional redundancy of tandem duplicated genes in allotetraploid cotton. Although there was no direct molecular evidence for the functional divergence of homoeologous gene pairs, the results of this study provide a whole genome information for research into gene fates in *G. hirsutum* during evolution and domestication.

## Conclusions

We performed the first genome-wide analysis of the *AP2/EREBP* family genes in cotton and conducted a detailed investigation of their classification, structure, gene expansion, synteny and expression profiles in different tissues and in response to various abiotic stresses. Results revealed that the *AP2/EREBP* genes were obviously expanded in *Gossypium* mainly by segmental duplication except for the B3 group of ERF subfamily mainly by tandem duplication. The *GhDREB* and *GhERF* genes play crucial roles in cotton stress responses. To the best of our knowledge, our data provide insights into characteristics and potential functions of cotton AP2/EREBPs. The results provide a useful basis for further research into the structure, function and phylogenetic relationships of these gene family members. This will help the identification of excellent candidate genes for genetic engineering to improve stress tolerance and developmental research in cotton and other valuable plants.

## Methods

### Identification and chromosomal mapping

The *G. raimondii*, *A. thaliana*, *V. vinifera*, and *T. cacao* gene files were downloaded from Phytozome v11.0 (http://www.phytozome.net/). The gene information of *G. hirsutum* acc. TM-1 was downloaded from http://mascotton.njau.edu.cn . The Hidden Markov Model (HMM) profile of the AP2 domain (PF00847) was obtained from the Pfam website (http://pfam.xfam.org/), and was employed as a query to identify all possible AP2/EREBPs using HMMER (V3.0) software [65]. To validate the HMM search, all candidate sequences were used as queries to search the NCBI non-redundant (nr) protein database with the blastp program, and the results with the best ‘AP2’ hits were retained for further analysis. To predict the GPI-anchor attachment sites, the BGI-PI47 [66] and GPI-SOM48 [67] algorithms were used. The AP2/ERF sequences were confirmed based on the presence of an AP2 domain, and all of the putative AP2/ERF proteins were aligned to Arabidopsis AP2/ERF proteins to classify them into different groups, as described by Feng et al. [20].

Positional information on all of the AP2/EREBPs was parsed from the General Feature Format (GFF) files downloaded from Phytozome v11.0, and the locations of AP2/EREBPs in *G. raimondii* and *G. hirsutum* were drafted using MapInspect software (http://mapinspect.software.informer.com/).

### Phylogenetic and gene structure analysis

A multiple alignment of the sequences encoding the conserved AP2/ERF domain was constructed with ClustalX (version 2.0) [68], and the gaps and poorly aligned sections were removed. A phylogenetic tree was generated using the maximum likelihood method and WAG model in MEGA v5.2 [69] software, and the reliability of interior branches was assessed with 1000 bootstrap resamplings.

The gene structures of the AP2/EREBPs were parsed from the General Feature Format (GFF) files, and diagrams of the exon-intron structures were drawn using the online program Gene Structure Display Server (GSDS; http://gsds.cbi.pku.edu.cn/).

### Gene duplication and synteny analysis

The syntenic information of *G. raimondii*, *A. thaliana*, *V. vinifera* and *T. cacao* was downloaded from the Plant Genome Duplication Database (PGDD; http://chibba.agtec.uga.edu/duplication/). AP2/EREBPs were mapped to the syntenic blocks for intra- and inter-genomic comparison. A syntenic diagram was drawn using Circos software [70].

The timing of segmental duplication events can be estimated by computing mean *Ks* values for all anchor points located in the corresponding syntenic block [43, 47], and all the *Ks* values were parsed from PGDD syntenic data. Genes separated by five or fewer genes within a 100-kb region on a chromosome may have resulted from tandem duplication [71].

### Plant materials and stress treatments

The widely used genetic standard line, *G. hirsutum* acc. Texas Marker-1 (TM-1), which is not conflict to any permissions or licences, was used for tissue/organ expression analysis. Roots, stems and leaves were collected from 2-week-old seedlings grown in a growth chamber. Petals, anthers and ovules were collected from plants grown under standard field conditions on the day of flowering, and fibers were excised from developing bolls on selected days post anthesis (dpa). True leaves of the seedlings were treated with PEG, heat and cold. All the RNA-Seq data were taken from Zhang et al. [42].

*G. hirsutum* acc. TM-1 was used to validate the expression profiles of the AP2/EREBPs under stress treatments. Cotton seedlings were grown in a growth chamber with fixed chamber condition (light/dark cycle: 14 h at 28 °C/10 h at 25 °C; 70% relative humidity). Three-week-old seedlings were treated as follows: for the drought stress, the roots of cotton seedlings were irrigated with 20% PEG), and for the temperature stress, seedlings were placed in a growth chamber at a low temperature conditions (4 °C). Seedlings grown in normal conditions were used as a mock control. The first two true leaves were collected at 0 h (just before stress treatment), and 1, 3, 6, 12 and 24 h after stress treatment,and immediately frozen in liquid nitrogen and stored at −70 °C. The experiments were repeated three times, each with 18 plants per treatment and standard errors were from the means of three biological replicates.

### RNA isolation and quantitative reverse transcription PCR

Total RNA was isolated using a plant RNA purification kit (MoLFarming, Cat.No. RK16-50 T, China) from leaf tissues according to the manufacturer's instructions. *T*-Test was used for statistical analysis. The expression of *GhDREB/ERFs* was analyzed using an ABI 7500 real-time PCR system with the SYBR Green Master Mix (Vazyme, Nanjing, China). Gene-specific primers were designed based on the *GhDREB/ERF* gene sequences using Primer Premier 5.0. Cotton histone3 (AF024716) was used as the reference gene [72]. The amplification parameters were as follows: 95 °C hold for 10 min, followed by 40 cycles at 95 °C for 15 s, 58 °C for 15 s and 72 °C for 15 s. For the melting curve stage, the default settings were chosen. Nonspecific products were identified by inspecting melting curves. All the primers used in this paper have been listed in an additional table: Additional file 14: Table S5.

### Investigation of the expressions pattern of *GhAP2/EREBP* genes

Expression data for *GhAP2/EREBP* genes was obtained from the transcriptome data [42]. These datasets correspond to gene expression intensities in various tissues and under abiotic stresses. Gene expression levels in the different tissues were calculated according to FPKM values and the default empirical abundance threshold of FPKM > 1 was used to identify the expressed genes. In abiotically stressed plants, genes with expression levels (FPKM) twofold or more greater than in controls were identified as up-regulated genes, and genes with expression levels that were less than one half of that in controls were identified as down-regulated. Expression patterns were clustered by Mev4.6.2 software using the Hierarchical Clustering model (http://www.tm4.org/mev.html).

## Additional files

Additional file 1: Table S1. AP2/EREBP superfamily genes in *G. raimondii*. (XLS 46 kb)
Additional file 2: Table S2. AP2/EREBP superfamily genes in *G. hirsutum* acc. TM-1. (XLS 77 kb)
Additional file 3: Figure S1. Gene clustering of the AP2/EREBP superfamily homologous genes in *G. raimondii* and *G. hirsutum*. Maximum likelihood method was used to the sequence alignment and phylogeny tree construction. (PDF 950 kb)
Additional file 4: Figure S2. Comparison of deduced amino acid sequences of the DREB and ERF conserved domains. The black background represents conserved amino acid residues in each group. (TIF 1648 kb)
Additional file 5: Figure S3. Phylogeny tree of the AP2/EREBP superfamily genes in *G. raimondii* and *A. thaliana*. (TIF 1028 kb)
Additional file 6: Table S3. Tandem duplicated genes of AP2/EREBP superfamily genes in *G. raimondii*. (XLS 33 kb)
Additional file 7: Figure S4. Gene structures of *GrAP2/EREBP* genes. Exons and introns are represented by blue boxes and black lines, respectively, and their sizes are indicated by the scale at the top. (TIF 477 kb)
Additional file 8: Table S4.
*Ks* values for segmental duplicated gene pairs of AP2/EREBP superfamily genes in *G. raimondii*. (XLS 60 kb)
Additional file 9: Figure S5. Synteny comparison of AP2/EREBP subfamily of *G. raimondii* to cacao (a) and grape (b). (TIF 4306 kb)
Additional file 10: Figure S6. Comparison number of *GrDREB*, *GrERF*, *GrAP2* and *GrRAV* genes among *G. raimondii*, *T. cacao* and *V. vinifera*. (TIF 58 kb)
Additional file 11: Figure S7. Venn diagram of transcripts identified in cotton under different abiotic stress conditions. Cold, drought and heat. (TIF 48 kb)
Additional file 12: Figure S8. Comparisons of expression profiles of nine representative homoeologous gene pairs of AP2/EREBP family of *G. hirsutum* in various tissues. Represented in y-axes are the FPKM levels of the RNA-seq data [42] and the x-axes are ten representative tissues. (TIF 189 kb)
Additional file 13: Figure S9. Expression profiles (in log2 based fold change) of tandem duplicated genes in *G. hirsutum* TM-1. Each block indicates one tandem duplicated gene cluster. The scale bars represent log2 of the RPKM values. (TIF 323 kb)
Additional file 14: Table S5. All the primers used in this paper. (XLSX 10 kb)

## Abbreviations

AP2: APETALA2
AP2/EREBP: APETALA2/ethylene-responsive element binding protein
DREB: Dehydration-Responsive Element Binding protein
ERF: Ethylene-Responsive Factor
Gh: *Gossypium hirsutum*
Gr: *Gossypium raimondii*
PGDD: Plant Genome Duplication Database
qRT-PCR: Quantitative reverse transcription PCR.
RAV: Related to ABI3/VP1
WGD: Whole-genome duplication
