# Supplementary figures and images for: Expansion and stress responses of the AP2/EREBP superfamily in cotton
Source: BMC Genomics. 2017 Jan 31;18:118. doi: 10.1186/s12864-017-3517-9 (PMC5282909; doi:10.1186/s12864-017-3517-9)

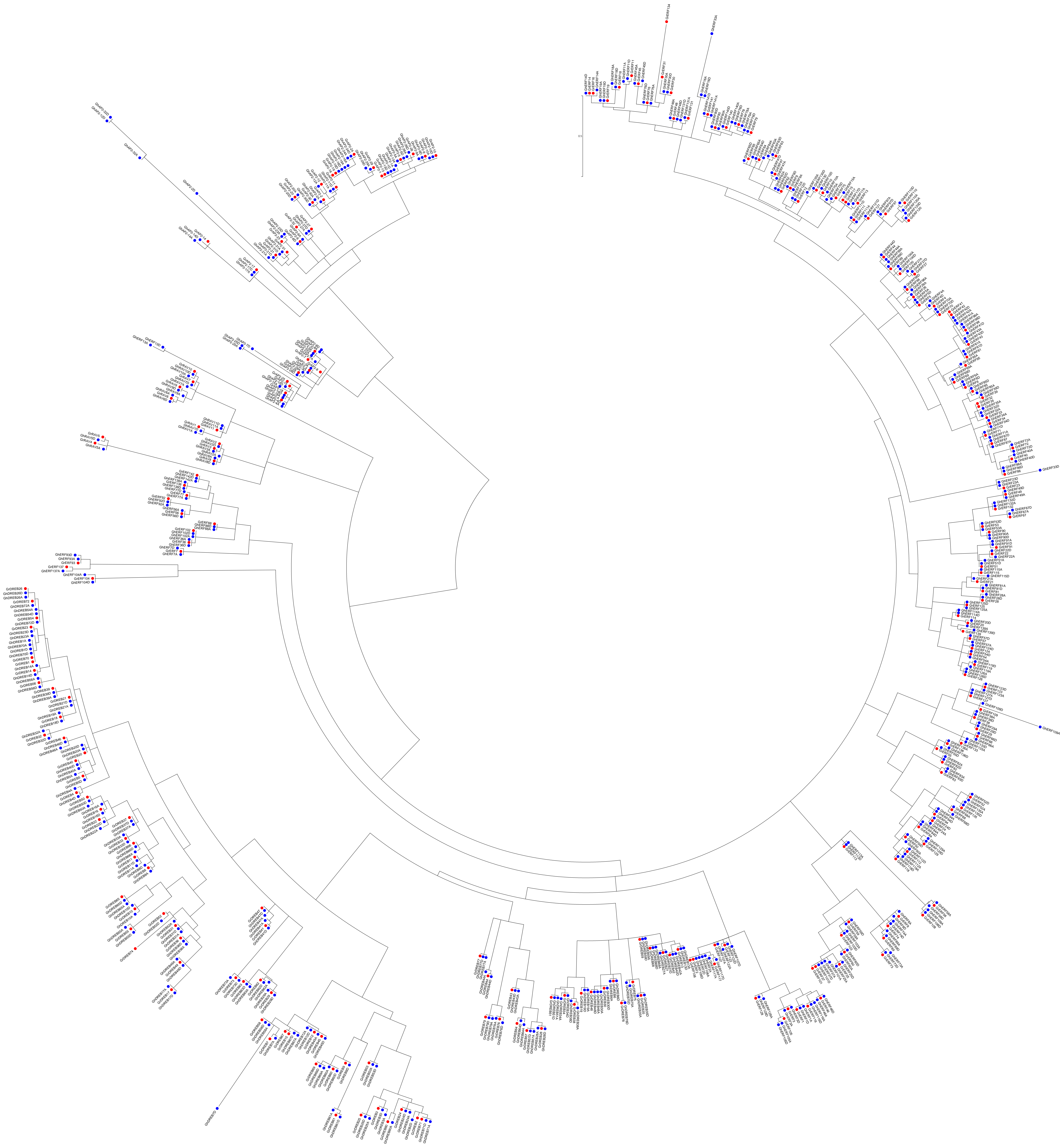

Supplement: Additional file 3: Figure S1. — Gene clustering of the AP2/EREBP superfamily homologous genes in G. raimondii and G. hirsutum. Maximum likelihood method was used to the sequence alignment and phylogeny tree construction. (PDF 950 kb) [file 12864_2017_3517_MOESM3_ESM.pdf]

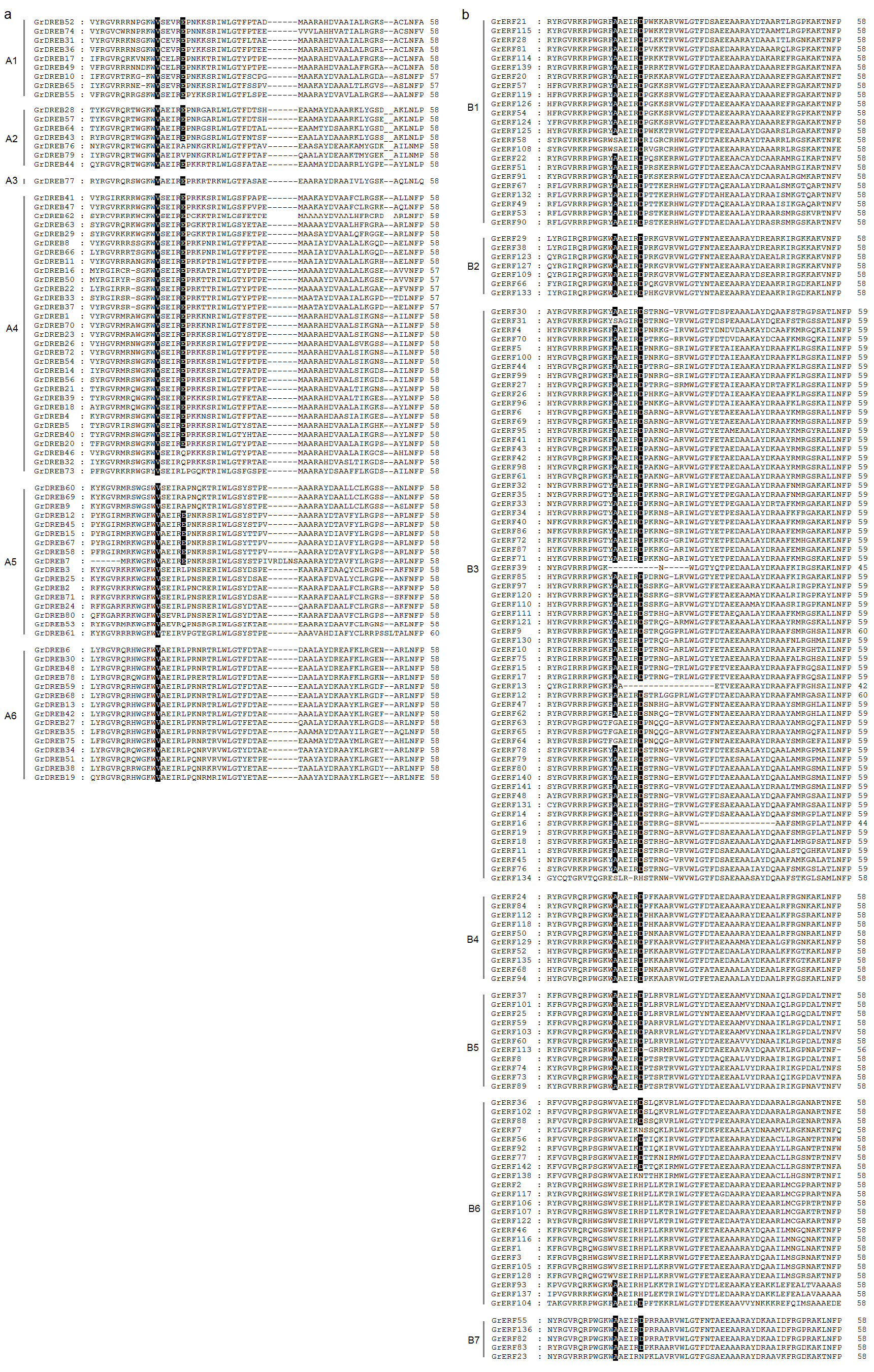

Supplement: Additional file 4: Figure S2. — Comparison of deduced amino acid sequences of the DREB and ERF conserved domains. The black background represents conserved amino acid residues in each group. (TIF 1648 kb) [file 12864_2017_3517_MOESM4_ESM.tif]

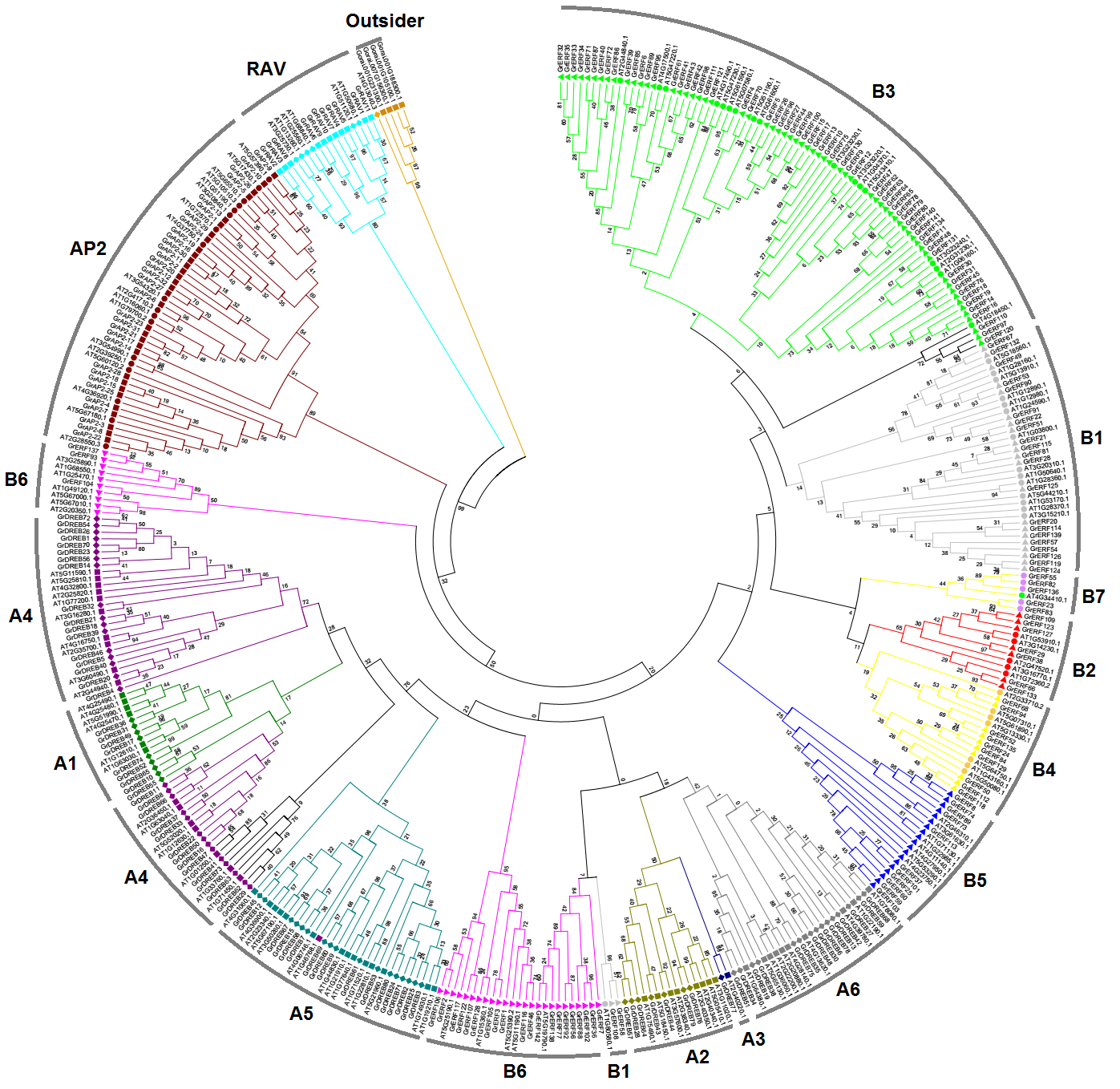

Supplement: Additional file 5: Figure S3. — Phylogeny tree of the AP2/EREBP superfamily genes in G. raimondii and A. thaliana. (TIF 1028 kb) [file 12864_2017_3517_MOESM5_ESM.tif]

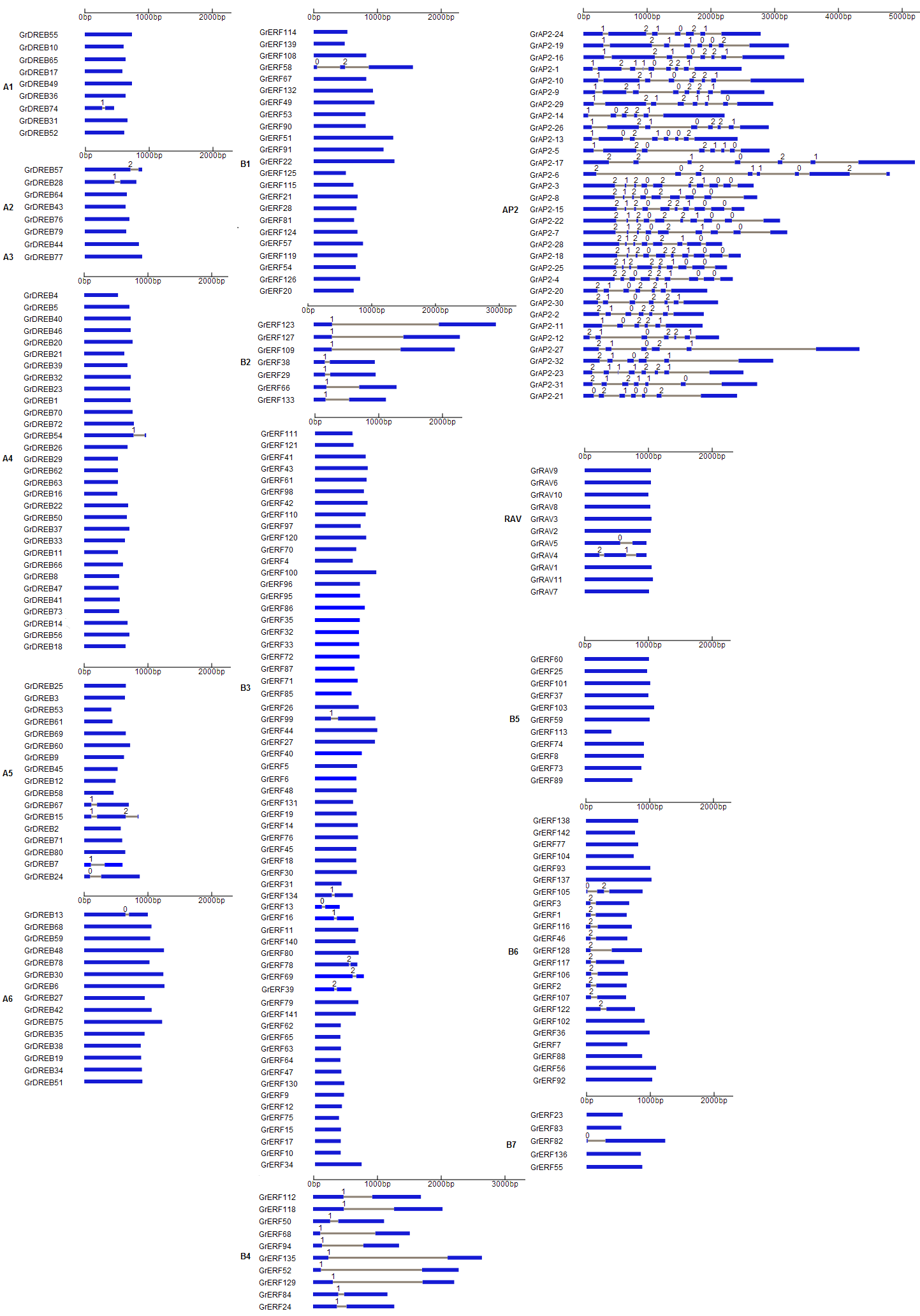

Supplement: Additional file 7: Figure S4. — Gene structures of GrAP2/EREBP genes. Exons and introns are represented by blue boxes and black lines, respectively, and their sizes are indicated by the scale at the top. (TIF 477 kb) [file 12864_2017_3517_MOESM7_ESM.tif]

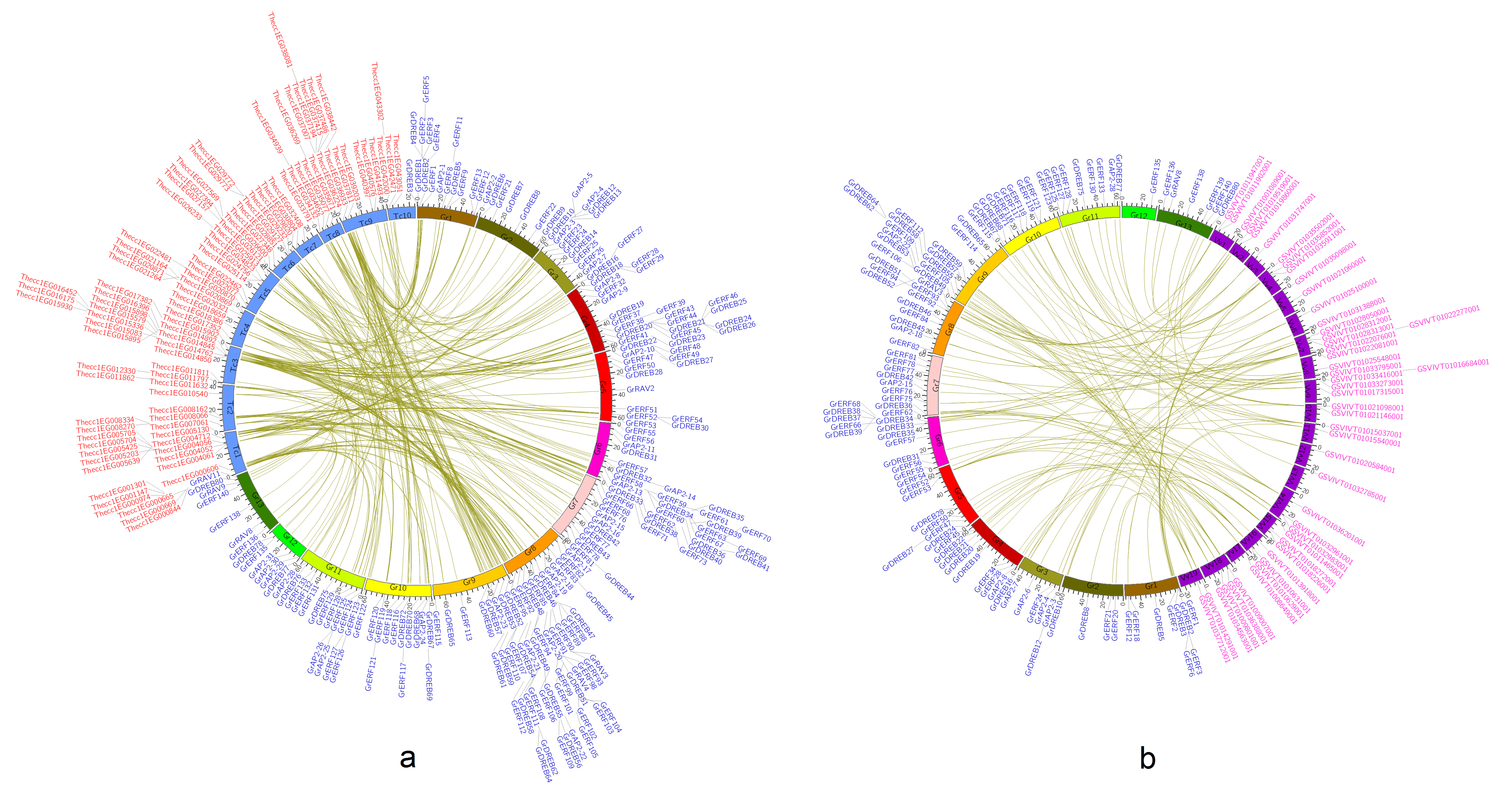

Supplement: Additional file 9: Figure S5. — Synteny comparison of AP2/EREBP subfamily of G. raimondii to cacao (a) and grape (b). (TIF 4306 kb) [file 12864_2017_3517_MOESM9_ESM.tif]

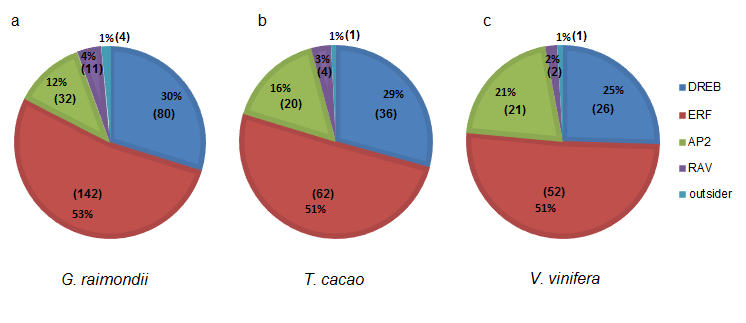

Supplement: Additional file 10: Figure S6. — Comparison number of GrDREB, GrERF, GrAP2 and GrRAV genes among G. raimondii, T. cacao and V. vinifera. (TIF 58 kb) [file 12864_2017_3517_MOESM10_ESM.tif]

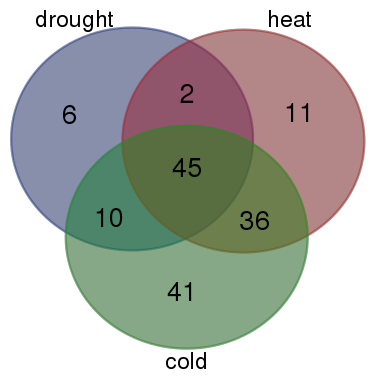

Supplement: Additional file 11: Figure S7. — Venn diagram of transcripts identified in cotton under different abiotic stress conditions. Cold, drought and heat. (TIF 48 kb) [file 12864_2017_3517_MOESM11_ESM.tif]

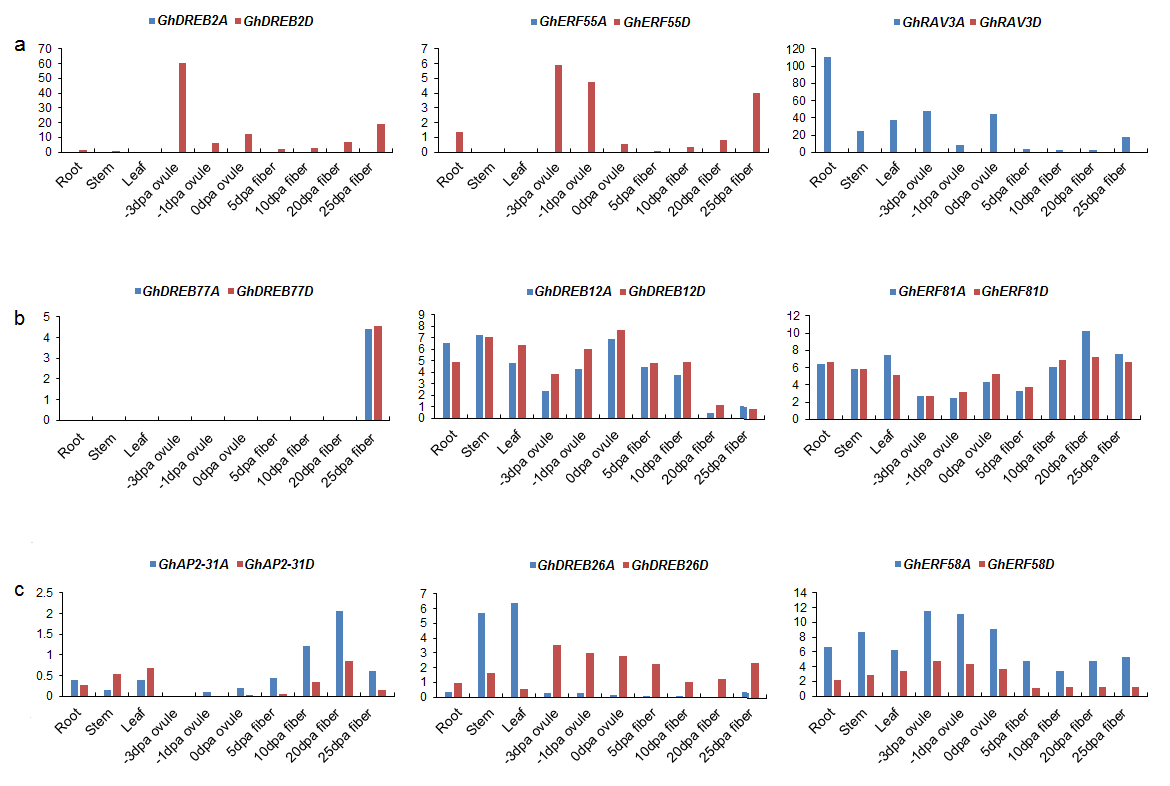

Supplement: Additional file 12: Figure S8. — Comparisons of expression profiles of nine representative homoeologous gene pairs of AP2/EREBP family of G. hirsutum in various tissues. Represented in y-axes are the FPKM levels of the RNA-seq data [42] and the x-axes are ten representative tissues. (TIF 189 kb) [file 12864_2017_3517_MOESM12_ESM.tif]

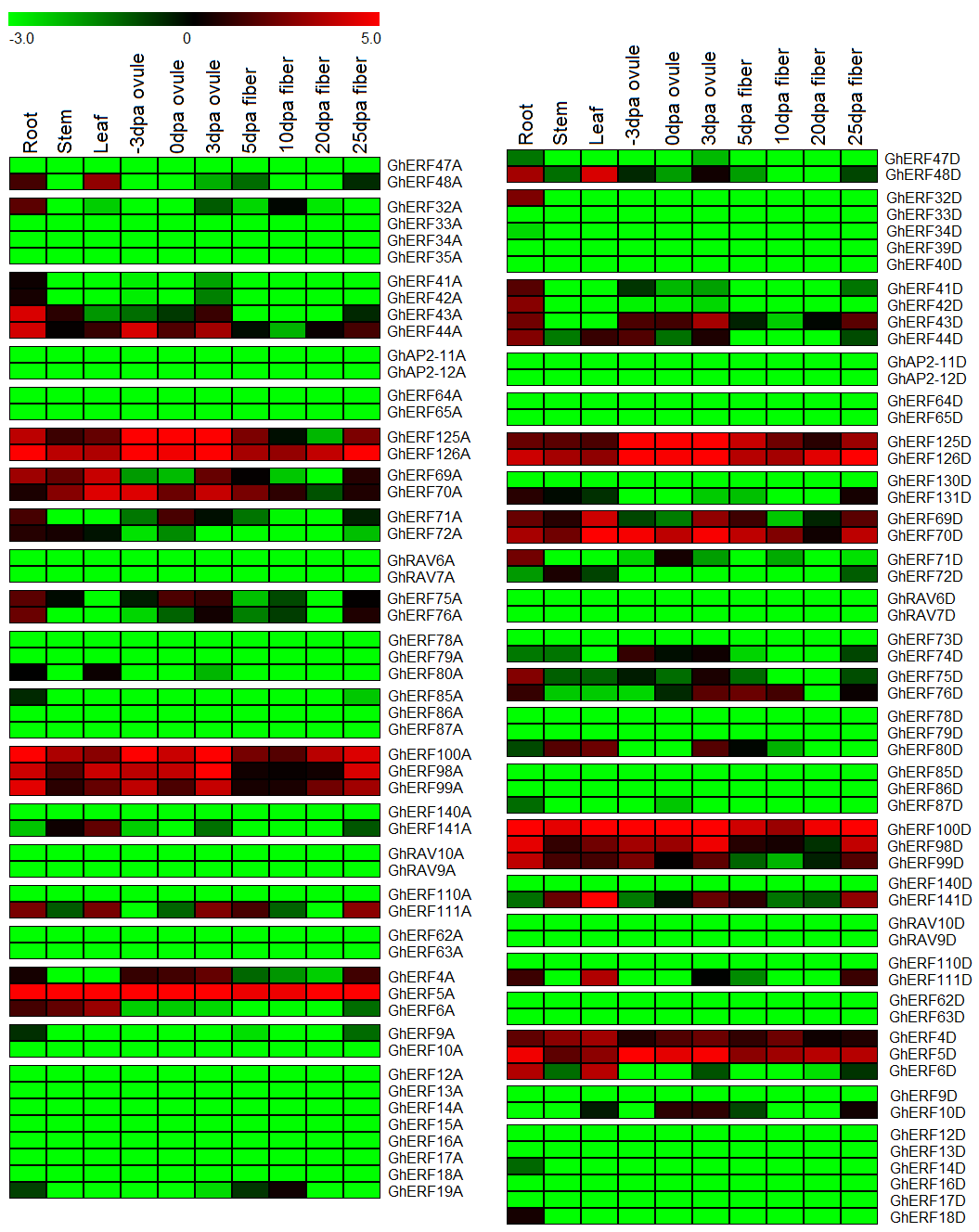

Supplement: Additional file 13: Figure S9. — Expression profiles (in log2 based fold change) of tandem duplicated genes in G. hirsutum TM-1. Each block indicates one tandem duplicated gene cluster. The scale bars represent log2 of the RPKM values. (TIF 323 kb) [file 12864_2017_3517_MOESM13_ESM.tif]
